# Thalamic atrophy in multiple sclerosis is associated with tract disconnection and altered microglia

**DOI:** 10.1007/s00401-025-02893-4

**Published:** 2025-05-28

**Authors:** Carla Rodriguez-Mogeda, Ismail Koubiyr, Stefanos E. Prouskas, Margarita Georgallidou, Susanne M. A. van der Pol, Rosalia Franco Fernandez, Yvon Galis de Graaf, Ysbrand D. van der Werf, Laura E. Jonkman, Geert J. Schenk, Frederik Barkhof, Hanneke E. Hulst, Maarten E. Witte, Menno M. Schoonheim, Helga E. de Vries

**Affiliations:** 1 Molecular Cell Biology and Immunology, Amsterdam UMC, Vrije Universiteit Amsterdam, De Boelelaan 1117, Amsterdam, The Netherlands; 2 MS Center Amsterdam, Amsterdam UMC Location VUmc, Amsterdam Neuroscience, Amsterdam, The Netherlands; 3 Anatomy and Neurosciences, Amsterdam UMC, Vrije Universiteit Amsterdam, De Boelelaan 1117, Amsterdam, The Netherlands; 4 Radiology and Nuclear Medicine, Amsterdam UMC, Vrije Universiteit Amsterdam, De Boelelaan 1117, Amsterdam, The Netherlands; 5 Queen Square Institute of Neurology and Centre for Medical Image Computing, University College London, London, WC1E 6BT UK; 6 Institute of Psychology, Health, Medical and Neuropsychology Unit, Leiden University, Wassenaarseweg 52, Leiden, The Netherlands

**Keywords:** Multiple sclerosis, Thalamus, Atrophy, Post-mortem, Microglia

## Abstract

Thalamic atrophy already occurs in the early stages of multiple sclerosis (MS) and continues progressively throughout the disease. Demyelination is one of the main pathological hallmarks of MS and yet, thalamic demyelination does not correlate well with thalamic atrophy. By combining post-mortem magnetic resonance imaging with immunohistochemistry of thalami from 13 control and 13 MS donors, we investigated the underlying pathological contributors of thalamic atrophy and pathology. We first assessed the volumes of four thalamic nuclei groups (anterior, lateral, medial and posterior). Then, diffusion weighted imaging was used to assess the microstructural integrity of white matter tracts connecting each thalamic nuclei group. In addition, we studied myelination, inflammation, neurodegeneration and microglial activation by immunohistochemistry. We uncovered that medial and posterior thalamic nuclei were more atrophic compared to the anterior and lateral nuclei. Bilateral posterior nuclei and the right medial and anterior nuclei showed reduced fractional anisotropy in connected white matter tracks. We further show that microglial cells in the mediodorsal nuclei have an increased density and morphological complexity in MS compared to control donors. Microglia show signs of phagocytosis of pre-synapses, although we did not observe an overall synaptic loss in the thalamus of MS donors. These microglial changes within mediodorsal nuclei correlated with lower medial thalamic volume. Taken together, this study provides evidence that thalamic (subnuclear) atrophy relates tostructural thalamic network disconnection and within-thalamic microglial changes, but not thalamic demyelination. These findings could impact future treatment strategies aimed at thalamic neuroprotection.

**Supplementary Information:**

The online version contains supplementary material available at 10.1007/s00401-025-02893-4.

## Introduction

Multiple sclerosis (MS) is a chronic neuroinflammatory and neurodegenerative disease that affects the CNS [12]. Demyelination is one of the main pathological hallmarks of MS, most typically visible on T2-weighted magnetic resonance imaging (MRI) as white matter lesions [12]. The clinico-radiological paradox describes the mismatch between what is visible on conventional MRI techniques and clinical symptoms [4]. This paradox is thought to be driven by additional underlying pathological mechanisms [25]. One important mechanism centers around neurodegeneration in the form of atrophy, captured on T1-weighted MRI, especially of (deep) grey matter structures like the thalamus, which is thought to drive cognitive and disability progression even in the absence of relapses or new lesions [6, 14, 16, 37].

Recent work has shown that atrophic grey matter regions most strongly related to clinical progression are usually brain network hubs [19]. These are brain regions that are highly connected. As such, MS is increasingly being recognized as a network disease, in which the breakdown of connections between different brain regions plays a central role in the development and progression of neurodegeneration [38]. In driving such pathological processes, this network disruption is thought to lead to a so-called “network collapse”, a threshold beyond which disconnection leads to clinical disability [38]. Of the network hubs shown to be degenerating in MS, the most clinically relevant is the thalamus [32]. Indeed, the thalamus is one of the first brain regions to show atrophy in the early stages of the disease with continuous progressive tissue loss throughout the disease [10]. Thus, thalamic volume is thought to represent a “barometer” of overall network disruption and hence overall disease burden in the brain, and is commonly used as a clinical trial endpoint [23, 35, 39]. Current research suggests that thalamic lesions are not strongly related to thalamic volume loss and that perhaps a white matter (WM) network disconnection syndrome might drive thalamic neurodegeneration [27, 29, 31, 49].

Furthermore, recent research has also highlighted the role of cerebrospinal fluid (CSF)-mediated factors in thalamic atrophy [28]. Neurotoxic factors within the CSF, such as proinflammatory and cytotoxic cytokines could diffuse into adjacent thalamic nuclei and trigger a cascade of degenerative changes, including demyelination, microglia activation and neuronal death [7]. Hence, thalamic nuclei closer to the ventricles could be more vulnerable to pathology due to a possible two-hit phenomenon, comprising Wallerian degeneration and toxic factors from the CSF [24]. Still, the involvement of thalamic microglia in thalamic atrophy has not been studied previously. Microglia are continuously scanning their environment for brain injuries and for debris clearance [34]. However, microglia have dichotomous roles in MS: they are crucial for maintaining neuronal networks and restoring brain homeostasis, but they can actively contribute to neuroinflammation and neurodegeneration [40, 48].

Despite the high clinical relevance and abovementioned hypothesis of underlying network disconnections, histopathological mechanisms driving MRI-measured thalamic pathology in MS remains understudied. This lack of knowledge and confirmation of the hypotheses related to network disconnection and proximity to CSF is especially driven by a need for histopathological data also including healthy control tissue and WM network imaging. In this study, we, therefore, interrogated the atrophy of thalamic nuclei in MS using in-situ post-mortem MRI scans in concert with immunohistochemistry (IHC) on the same tissues. We examined volumetric MRI differences of thalamic nuclei in post-mortem MS donors compared to controls, and assessed microstructural integrity of connected WM tracts. Using IHC of thalamic blocks from the same donors, we studied how intra-thalamic inflammation and microglial changes, including microglia density, morphology and phagocytosis of pre-synapses, contribute to thalamic atrophy.

## Materials and methods

### Subjects and tissue collection

In this study, we included 13 cases with confirmed progressive MS and 13 pathologically confirmed non-neurological controls. Shortly after death, MRI in MS cases was performed and tissue was collected through the standardized MS center Amsterdam rapid autopsy protocol in collaboration with the Netherlands Brain Bank (NBB) [5]. Healthy volunteer data collection followed similar protocols according to protocols of the Normal Aging Brain Collection Amsterdam (NABCA) [22]. Prior to death, all subjects had registered with the NBB or full body donation program at the department of Anatomy and Neurosciences at Amsterdam UMC, providing informed consent for autopsy and use of imaging, tissue and medical records for research purposes. Permission for the protocols was provided by the institutional ethics review board. Clinical and demographic information is summarized in Table 1. In more detail, 92.3% of the MS cases were female, with a mean age and standard deviation at time of death of 63.76 ± 12.10 and a disease duration of 26.54 years ± 0.04; 61.5% of the pathologically confirmed non-neurological controls were female, with a mean age and standard deviation at time of death of 70.38 ± 6.64. For both groups, the most common cause of death was euthanasia and lung complications (e.g., infections, failure). After MRI scanning, formalin-fixed paraffin-embedded (FFPE) blocks of the thalamus were cut into 5 and 10μm sections and stored at room temperature until further use.

**Table 1:** Clinical data of the cohort

ID	Disease status	Sex	Age	Disease duration (years)	Post-mortem delay (h:m)	Cause of death
MS1	progMS	f	52	27	8:40	Euthanasia
MS2	progMS	f	75	25	10:30	Sepsis
MS3^a^	progMS	f	65	16	10:45	Cerebrovascular accident
MS4	progMS	f	62	31	8:40	Euthanasia
MS5	progMS	f	40	8	8:30	Multiple sclerosis, coma
MS6	progMS	f	77	27	8:20	Respiratory insufficiency
MS7	progMS	f	81	51	7:20	Dehydration
MS8	progMS	m	75	42	9:05	Euthanasia
MS9^b^	progMS	f	65	37	10:20	MS-induced pneumonia
MS10	progMS	f	52	17	9:10	Euthanasia
MS11	progMS	f	68	25	9:20	Pneumonia / urosepsis
MS12	progMS	f	51	13	10:15	Euthanasia
MS13^c^	progMS	f	66	26	9:30	Euthanasia
C1	Control	f	68	NA	5:00	Euthanasia
C2	Control	m	63	NA	6:35	Euthanasia
C3	Control	m	72	NA	5:45	Heart failure
C4	Control	f	69	NA	9:00	Pulmonary embolism
C5	Control	f	59	NA	6:15	Euthanasia
C6	Control	f	74	NA	15:00	Cancer
C7	Control	m	72	NA	10:30	Esophagus cancer, lung failure
C8	Control	f	77	NA	9:45	Pneumonia
C9	Control	f	79	NA	4:00	Unknown
C10	Control	f	78	NA	7:30	Unknown
C11	Control	f	59	NA	6:40	Euthanasia
C12	Control	m	71	NA	4:45	Lung Carcinoma
C13	Control	m	74	NA	8:45	Euthanasia

*h:m* hours:minutes, *f* female, *m* male, *NA* not applicable, *progMS* progressive multiple sclerosis

^a^Excluded from Fig. 4, 5, 6, Fig. S2-3

^b^Excluded from Fig. 3d, Fig. 6

^c^Excluded from Fig. 2b–d, Fig. 6

### Magnetic resonance imaging (MRI)

MRI was performed on a 3T whole-body scanner (GE MR750 Discovery) with an eight channel phased-array head coil, as previously described [5]. Imaging was performed both in-situ (with brain still in cranium) and of subsequently derived 1cm-thick coronal brain slices, from which thalamic sections were cut. By matching the slices with the in-situ 3D imaging, the precise anatomical locations of the thalami were determined. The imaging protocol consisted of a sagittal three dimensional (3D) T1-weighted fast spoiled gradient-echo sequence (TR = 6.7 ms; TE = 3 ms, TI = 450 ms, slice thickness = 1.0 mm, in-plane resolution = 0.98 × 0.98 mm^2^), a sagittal 3D T2-fluid-attenuated inversion recovery (FLAIR) scan (TR = 8000 ms; TE = 125 ms; TI = 2000ms; slice thickness = 1.2 mm, in-plane resolution = 0.98 × 0.98 mm^2^) and an axial two dimensional (2D) echo-planar imaging (EPI) pulse sequence with diffusion gradients applied in 30 non-collinear with b = 1000s/mm^2^, and five volumes acquired without diffusion weighting (TR = 8000 ms, TE = 85 ms, slice thickness = 2.0 mm, in-plane resolution = 2.0 × 2.0 mm^2^). 3D-FLAIR was used for white matter lesions segmentation while 3D-T1 was used for volumetry. In control donors, the few segmented lesions likely reflect age-related white matter changes. Smaller vascular-type lesions were not included, as they did not meet the minimum size threshold for MS lesion segmentation. These lesions were segmented primarily to improve the accuracy of T1-weighted image processing and avoid segmentation errors during volumetric analysis. Imaging parameters have been published previously [5, 22].

#### MRI processing: volumes

Lesions were segmented on 3D-FLAIR using a semi-automated technique and manual corrections. Lesion maps were then co-registered to 3D-T1 and used for lesion filling. Subsequently, AssemblyNet software was used to determine global brain volumes and deep grey matter volumes [9].

Thalamic nuclei segmentation was performed with a modified version of Thalamus Optimized Multi Atlas Segmentation (THOMAS) method [43], which uses T1-weighted (T1w) imaging as an input image to segment the thalamus [47]. This method specifically uses two separately trained convolutional neural networks (CNN) to synthesize white matter-nulled (WMn)-MPRAGE images from T1w data and then segment the synthetic data based on the histological definitions of the Morel atlas [33]. The analysis results in the segmentation of 10 thalamic nuclei, which were then grouped into four main regions based on their anatomical and functional location: anterior group (anteroventral: AV; ventral anterior: VA), lateral group (ventral posterolateral: VPL; ventral lateral anterior: VLa; ventral lateral posterior: VLp), medial group (mediodorsal: MDn; centromedian: CM) and posterior group (pulvinar: Pul; medial geniculate nucleus: MGN; lateral geniculate: LGN). Each group was blindly checked and manually corrected if needed. For the volumetric analysis of all participants, the volumes of the right and left sides were summed and assessed as a fraction of the volume of the intracranial cavity (IC) extracted through AssemblyNet to account for variations in head size [9].

#### Tractography and diffusion tensor imaging

Probabilistic tractography was performed using MRtrix3 software (http://www.mrtrix.org) [45]. A whole-brain mask was created for each subject after the diffusion data had been corrected for eddy current distortions and motion artefacts. Diffusion tensors were also generated from the whole brain mask, from which fractional anisotropy (FA) and mean diffusivity (MD) maps were obtained with tensor2metric. Using the ss3t_csd_beta1 function of MRtrix3Tissue (https://3Tissue.github.io), a fork of MRtrix3, a single-shell response function was calculated to estimate the Fibre Orientation Distributions (FOD) based on constrained spherical-deconvolution [46]. Ten million whole-brain tracts were produced using five tissue-type segmented T1 images and anatomically constrained tractography [45]. To reduce reconstruction bias and increase biological plausibility, these streamlines were ultimately filtered to one million using the spherical-deconvolution-informed filtering of tractograms [42]. We also assessed the microstructural integrity of each thalamic nuclei group using both FA and MD.

#### Construction of connectivity atlas and structural connectomes

FreeSurfer (v5.3) (http://surfer.nmr.mgh.harvard.edu/) was used for whole-brain segmentation using T1-weighted images [11]. To construct a connectivity matrix for our analysis, the Destrieux cortical atlas was used as the basis for our custom-made atlas [13]. The cortical atlas was registered to diffusion space, followed by the registration of the T1-image of segmented thalamic groups to diffusion B0 space using the flirt function of FSL [3]. All registrations were checked visually. The registered Destrieux cortical atlas was then modified to maintain only labels of interest, and thalamic groups were added to the image to create a custom-made atlas. Finally, the personalized atlas featured 172 regions.

The 1 million streamlines previously obtained were mapped into the custom atlas, and a 172 × 172 structural connectivity matrix was produced. Each matrix element reflected the number of streamlines for each subject. The structural connectivity matrix was then normalized by the maximum number of tracts for each participant, to bring the connectivity values within a consistent range. Connectivity matrices were also weighted by the mean values of FA per streamline.

### Immunolabelling

Thalamic sections were deparaffinized in xylene and rehydrated in a series of graded ethanol (100%, 90%, 80% and 70% for 3 min each). For staining for Nissl bodies, sections were stained with Thionin (Thermo Fisher Scientific) directly afterwards and mounted with Entellan. For all the other sections, after a rinse in milliQ, epitope retrieval was performed in citrate buffer pH 6 for 30 min in a water bath at 95ºC. Sections were cooled on ice and washed with PBS, followed by a blocking step with a PBS solution containing 10% normal serum (from the host of the secondary antibody) and 0.05% Tween-20 (Sigma) for 30 min at room temperature. Sections were incubated overnight at 4ºC with primary antibodies (Table 2) diluted in 3% normal serum and 0.05% Tween-20.

**Table 2:** Primary antibody details

Antigen	Clone	Species	Dilution	Antigen retrieval	Manufacturer	Cat. number
CD3	F7.2.38	Mouse	1:20	EDTA-Tris pH 8	Dako	M7254
CD19	EPR5906	Rabbit	1:100	EDTA-Tris pH 8	Abcam	Ab134114
HLA-DR	NA	Mouse IgG2b	1:500	Citrate pH 6.0	In house	NA
IBA1	Polyclonal	Goat	1:500	Citrate pH 6.0, EDTA-Tris pH 8	Abcam	Ab5076
LAMP1	D2D11	Rabbit	1:100	EDTA-Tris pH 8	Cell Signaling	9091P
HuC/HuD	16A11	Mouse	1:100	Citrate pH 6.0	ThermoFisher Scientific	A-21271
P2Y12	Polyclonal	NA	1:500	Citrate pH 6.0	Anaspec	55042A
PLP	Plpc1	Mouse	1:500	NA	Serotec	MCA839G
Synaptophysin	DAK-SYNAP	Mouse	1:500	EDTA-Tris pH 8	DAKO	M7315

^*NA* not applicable^

For immunohistochemistry, sections were subsequently incubated with EnVision + Dual Link System-HRP (Agilent Dako) with 3,3’-diaminobenzidine (DAB) as the chromogen. Sections were counterstained with hematoxylin, dehydrated in alcohol and xylene series, and embedded in Entellan medium (Merck). Sections were stored at room temperature until image acquisition. For immunofluorescence, primary antibody incubation was followed by a 2h incubation of Alexa fluorophore-labeled secondary antibodies at room temperature (1:400, Thermo Fisher Scientific). After washing with PBS, sections were incubated with DAPI (1:10,000, Molecular Probes) for nuclear visualization. Brain auto-fluorescence was quenched with 0.03% Sudan Black (Sigma) in 70% ethanol for 5 min. After washing, sections were mounted with Mowiol medium and stored at 4ºC in the dark until image acquisition.

### Microscopy image acquisition and image analysis

Bright-field images were taken using a 20 × objective on a Vectra Polaris whole-slide scanner (Akoya Biosciences). Fluorescent-labelled sections of microglia were imaged on a Vectra Polaris whole-slide scanner, a Nikon A1R laser-scanning confocal microscope, a Nikon AXR confocal microscope and a Leica TCS SP8 confocal microscope. Sections for synaptic markers were acquired on the Olympus VS200 slide scanner. Specific details for each staining are described in the sections below.

#### Mediodorsal nuclei location

The thalamus is divided into different nuclei and each nucleus has unique inputs and projections towards different cerebral cortical regions. Hence, the thalamus contains histologically a high heterogeneity depending on the specific nucleus [20]. To avoid misleading results, we decided to focus specifically on the mediodorsal nucleus (MDn), which is involved in cognition, emotion and attention, and it was present in all the blocks of our tissue cohort [15]. To pinpoint the different thalamic nuclei, we combined the autopsy images of the thalamus removal with the MRI post-processed images to define the anterior–posterior organization. We used bright field images for Nissl, PLP and synaptophysin to identify structures such as the choroid plexus or ependymal layers and to define the medial and lateral organization of the thalamic block. With the help of the neuroanatomy book [30], and by looking at the neuronal and synapse stainings, we identified the corresponding nuclei of each section.

#### Demyelination and neuronal loss

Whole slide scans of PLP stained sections were imported in ImageJ and MDn was outlined to calculate the surface area. All MDn lesions were manually annotated to calculate the respective area covered by lesions. These two areas were used to calculate the percentage of demyelinated MDn.

Per section, 3 confocal images for HuC/HuD / DAPI within the thalamic MDn were taken using a 20 × objective and a z-stepside of 0.5 µm on a Nikon AXR confocal microscope. To quantify neuronal density, these images were imported in QuPath (version 0.2.1). Cell detection function was used to count all DAPI^+^ nuclei. The numbers of HuC/HuD^+^DAPI^+^ neuronal cells were manually counted in QuPath. We determined the percentage of positive cells divided by the total number of DAPI^+^ nuclei and multiplied by 100 and the absolute count of neuronal cells divided by the total area of the images.

#### Mediodorsal adaptive immune cells

Fluorescent whole-slide images stained for CD3/CD19/DAPI were acquired with a 20 × objective on a Vectra Polaris scanner. Of the whole slides, we blindly selected 10–15 random areas of the MDn. These regions were saved as TIFF files using QuPath (version 0.2.1). NIS Elements software (version 5.42.01) was used to count all DAPI^+^ nuclei. The numbers of CD3^+^DAPI^+^ and CD19^+^ DAPI^+^ immune cells were manually counted in FIJI. We determined the percentage of positive immune cells divided by the total number of DAPI^+^ nuclei and multiplied by 100 and the absolute count of immune cells divided by the total area of the images.

#### Microglia density, protein expression and morphology

Per section, 5 confocal images for IBA1/P2Y12/DAPI within the thalamic MDn and 5 confocal images for IBA1/HLA-DR/DAPI within the thalamic MDn were taken using a 40 × objective and a z-stepside of 0.5 µm on a Nikon A1R confocal microscope and a Nikon AXR confocal microscope, respectively. In addition, 10 confocal images for IBA1/P2Y12/DAPI randomly distributed, regardless of MDn position, were taken in the whole thalamus using a 40 × objective and a z-stepside of 0.5 µm on a Nikon A1R confocal microscope.

For microglial density, NIS Elements software (version 5.42.01) was used to count all DAPI^+^ nuclei and IBA1^+^DAPI^+^ cells in the confocal images for IBA1/P2Y12/DAPI within the thalamic MDn and the images randomly distributed in the whole thalamus. The average count of the images was used for the following calculations: we determined the percentage of microglia as IBA1^+^DAPI^+^ cells divided by the total number of DAPI^+^ nuclei and multiplied by 100; we calculated the microglia density as IBA1^+^DAPI^+^ cells divided by the volume of the images.

For P2Y12 and HLA-DR protein expression, Imaris software (version 9.9.1, Bitplane AG) was used to segment all IBA1^+^ microglia using the surface function. From these surfaces, we measured the total fluorescence intensity sum of P2Y12 or HLA-DR divided by the total microglial volume. The final mean fluorescence intensity corresponded to the average of the 5 images.

For microglial morphology, we generated 2D maximum intensity projections of the aforementioned confocal images and manually traced IBA1^+^ P2Y12^+^ cells in a blind setting using FIJI as previously described [48]. We randomly selected microglia following this inclusion criteria: microglia should be included within the borders of the image, microglia should not overlap with any other cell and they should not be interacting with vessels. All traced cells were analysed using the Sholl Analysis Plugin [17] with a 0.3 µm step size from the cell soma. Per image, we analysed around 10 cells.

#### Pre-synaptic density and pre-synaptic microglial phagocytosis

Sections stained for IBA1/LAMP1/Synaptophysin/DAPI were acquired on a Leica TSC SP8 confocal microscope with a 100 × objective and a z-stepside of 0.1 µm. Per donor, 20 confocal images were taken in the MDn including one or two microglia in each image. We analysed pre-synaptic phagocytosis by microglial cells as previously described [48]. Briefly, we generated surfaces for DAPI^+^, IBA1^+^, LAMP1^+^ and IBA1^+^LAMP1^+^ signal with the Imaris software (version 9.9.1, Bitplane AG). Using the spot function with a spot diameter of 0.5µm and a quality filter, Synaptophysin^+^ pre-synapses were detected. All the steps were blindly individually set per donor. Spots in IBA1^+^ and IBA1^+^LAMP1^+^ surfaces were identified as spots that had a maximum distance of 0µm to these respective surfaces. Microglia phagocytosis was calculated by dividing the number of spots in IBA1^+^ and IBA1^+^LAMP1^+^ surfaces by the total number of spots or the total volume of the images. The volume of lysosomes in microglia was calculated as the average of the 20 images of the volume of LAMP1^+^ in IBA1^+^ surface.

To quantify pre-synaptic density, sections stained for Synaptophysin were scanned on an Olympus VS200 slide scanner. Specifically, 20 images per donor with a 60 × objective and a z-stepside of 0.25µm were acquired. Images were blindly taken in different areas of the MDn. The images were uploaded in Huygens software (version 23.04) to remove autofluorescence and deconvolve. Deconvolved images were uploaded in Imaris software (version 9.9.1) and using the spot function with a spot diameter of 0.5µm and a quality filter, Synaptophysin^+^ pre-synapses were quantified. Results are shown as the total number of pre-synapse spots divided by the total volume of the images and the percentage of the total volume occupied by pre-synapse spots divided by the total volume of the images.

### Statistics

For the MRI analysis, SPSS (version 28) was used for all statistical tests, while for the microscopy analysis, GraphPad Prism 9 was used. To assess the differences between control and MS donors across thalamic nuclei volumes, multivariate analysis of variance (MANCOVA) was conducted. Sex and post-mortem delay (time between death and in-situ MRI) were included in the model as covariates. To investigate whether cortical regions structurally connected to the MD nuclei of the thalamus also exhibit atrophy in MS, we identified corresponding frontal regions using FreeSurfer-based anatomical labels and tractography data from each subject. These included the anterior cingulate gyrus, middle anterior and middle posterior cingulate gyri, and the middle frontal gyrus, known to receive projections from the MD nuclei. We extracted the cortical volumes of these regions and performed a MANCOVA comparing MS and control donors, including sex and post-mortem delay to MRI as covariates. Effect sizes were calculated using Partial eta squared (η2). For microscopy analysis, we first tested for normality using Shapiro–Wilk to decide the appropriate statistical tests. Unpaired two-tailed Student’s t-test with or without Welch’s correction for unequal variances, or the non-parametric Mann–Whitney test were used accordingly. For correlations between MRI and histopathological variables, MRI data was selected from the same side as the thalamus block collected during autopsy for immunolabeling and partial Spearman’s correlations with post-mortem delay as a covariate were performed in SPSS. Data were considered significant when *P* < 0.05 and reported in the figures using the corresponding significance levels and statistical tests indicated in the figure legends. To plot the heatmaps of the r value from the correlations, we used the package *pheatmap* in R studio (version 4.2.1). Exclusion criteria from certain analysis was the absence of antibody reactivity or poor-quality diffusion measurements, as indicated in Table 1.

## Results

### Presence of thalamic atrophy in progressive MS

**Fig. 1:**
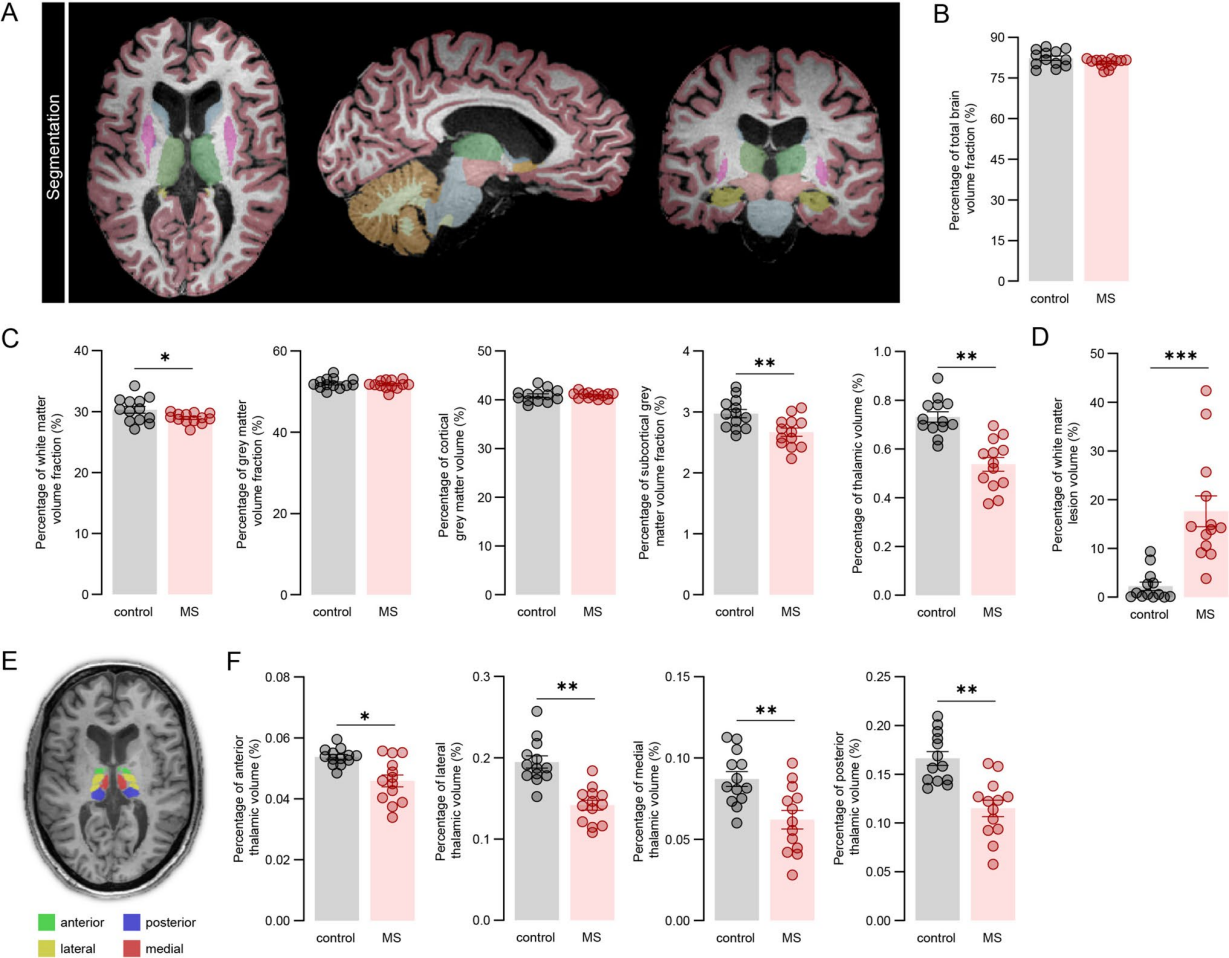
Increased atrophy in the thalamus of progressive MS donors. **a** Example of brain segmentation on 3D-T1 and 3D-FLAIR scans. **b** Normalized total brain volume of control and MS donors. **c** Percentage of different brain volume fractions of control and MS donors. **d** Percentage of white matter volume lesion of control and MS donors. **e** Example of thalamic segmentation on 3D-T1 and 3D-FLAIR scans. **f** Percentage of each thalamic nuclei volume of control and MS donors. Individual datapoints indicate data from an individual donor, columns and error bars show mean ± SEM; * *P* < 0.05, ** *P* < 0.01, *** *P* < 0.001. *MS* multiple sclerosis

While the MS group had similar total brain volume compared to control donors (Fig. 1a–b), MS donors showed lower global normalized white matter volume (mean volume fraction: MS = 28.939 vs. control = 30.289; *P* = 0.039; 4.3% reduction) and lower normalized subcortical grey matter volume (mean volume fraction: MS = 2.667 vs. control = 2.971; *P* = 0.008; 6.9% reduction, Fig. 1c). As expected, there was a lower normalized whole thalamic volume in MS donors (mean volume fraction: MS = 0.537 vs. control = 0.731; *P* = 0.001; 24.1% reduction), along with a higher white matter lesion volume (*P* < 0.001) (Fig. 1d). We further segmented the thalamic nuclei and grouped them into four anatomical groups: anterior, lateral, posterior, and medial (Fig. 1e). All four thalamic groups showed significantly reduced normalized volume in MS compared to control donors: anterior nuclei (mean volume fraction: MS = 0.046 vs. control = 0.054; *P* = 0.036; 13.7% reduction), lateral nuclei (mean volume fraction: MS = 0.142 vs. control = 0.195; *P* = 0.004; 25.0% reduction), posterior nuclei (mean volume fraction: MS = 0.115 vs. control = 0.166; *P* = 0.001; 27.5% reduction,), and medial nuclei (mean volume fraction MS = 0.062 vs. control = 0.087; *P* = 0.001; 25.9% reduction).

Medial and posterior nuclei were most atrophic as seen by the largest effect sizes (η2 = 0.361; η2 = 0.409, respectively) (Fig. 1f), i.e. thalamic nuclei closer to the CSF. We found no significant differences in the volumes of cortical regions connected to the MDn between MS and control donors. The normalized volumes (mean ± SD) and corresponding p-values were as follows: anterior cingulate gyrus – controls: 0.66 ± 0.07; MS: 0.67 ± 0.07 (*P* = 0.50), middle anterior cingulate gyrus – controls: 0.31 ± 0.03; MS: 0.31 ± 0.04 (*P* = 0.81), middle posterior cingulate gyrus – controls: 0.31 ± 0.05; MS: 0.31 ± 0.04 (*P* = 0.85), and middle frontal gyrus – controls: 1.34 ± 0.14; MS: 1.29 ± 0.13 (*P* = 0.89).

### Impaired structural connectivity in MS correlates with thalamic nuclei volumes

**Fig. 2:**
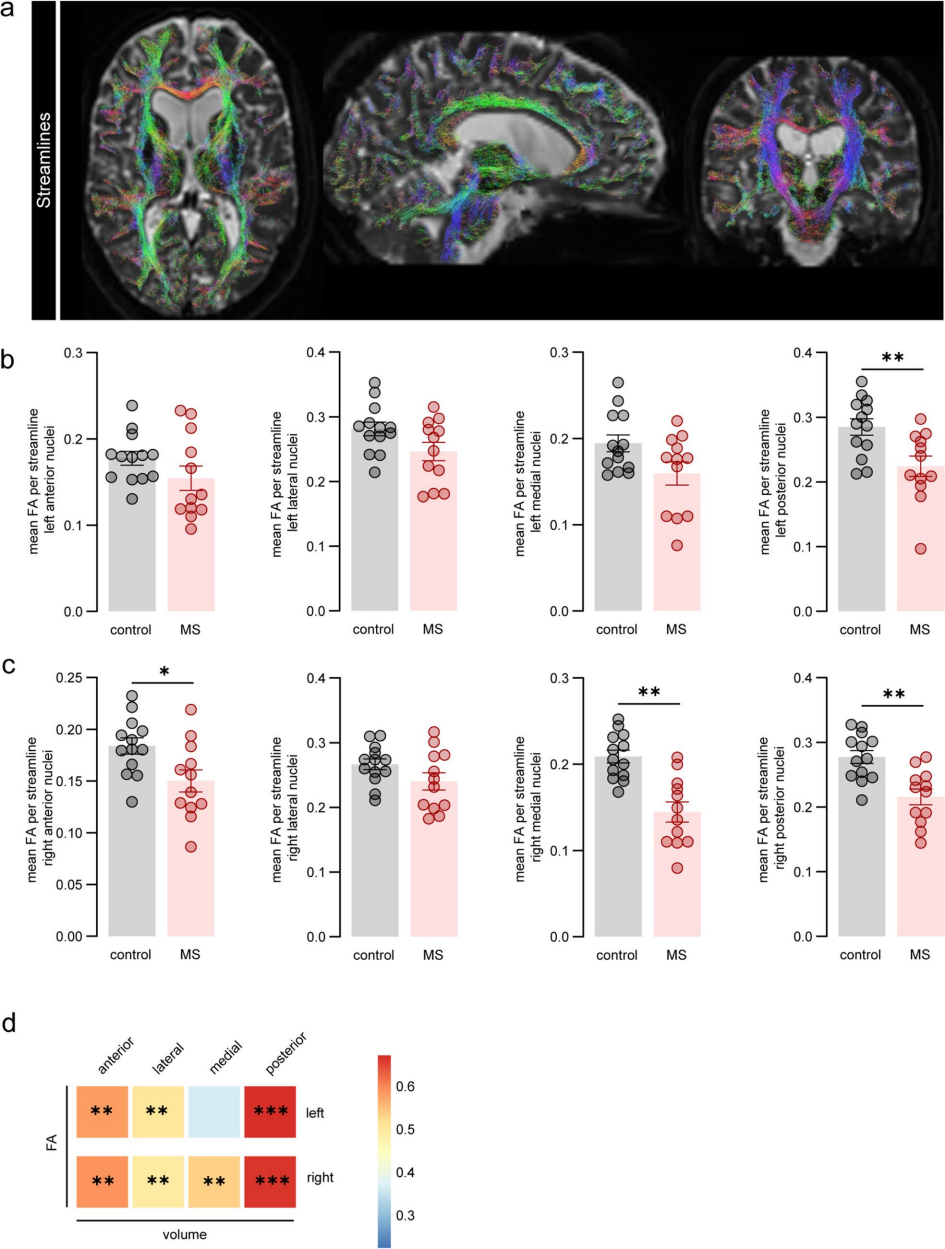
Increased disconnection in the thalamus of progressive MS donors. **a** Example of brain streamlines on 3D-T1 and 3D-FLAIR scans. **b** Mean FA per streamline for each left thalamic nuclei. **c** Mean FA per streamline for each right thalamic nuclei. **d** Heatmap indicating partial correlation values between mean FA and thalamic volume. Colour key represents high (red) and low (blue) positive correlations. Individual datapoints indicate data from an individual donor, columns and error bars show mean ± SEM; * *P* < 0.05, ** *P* < 0.01, *** *P* < 0.001. *FA* fractional anisotropy, *MS* multiple sclerosis

Next, we studied the microstructural integrity of the streamlines connecting the different thalamic nuclei to the cortex (Fig. 2a). Overall, the results from mean FA-weighted connectivity analysis showed reduced FA in thalamocortical tracts in MS donors compared to controls in the left (Fig. 2b) and right thalamus (Fig. 2c). In more detail, a significant FA reduction in tracts was found for the bilateral posterior nuclei (left: *P* = 0.010; right: *P* = 0.03) and right medial (*P* = 0.002) and anterior nuclei (*P* = 0.031). These changes indicate more disrupted or damaged WM tracts in MS donors and specifically, in thalamic nuclei close to the ventricles.

We then investigated the association between the mean FA of thalamocortical tracts and the volume of the corresponding thalamic nuclei (Fig. 2d). The FA of tracts connecting the bilateral anterior (left: *r* = 0.581, *P* = 0.003; right: *r* = 0.589, *P* = 0.014), lateral (left: *r* = 0.502, *P* = 0.012; right: *r* = 0.496, *P* = 0.014), posterior (left: *r* = 0.673, *P* < 0.000; right: *r* = 0.665, *P* < 0.000), and right medial nuclei (left: *r* = 0.368, *P* = 0.077; right: *r* = 0.536, *P* = 0.007) was significantly and positively correlated with a reduction in the corresponding thalamic nuclei volumes. Hence, reduced integrity of streamlines was associated with decreased thalamic volumes. Furthermore, we did not observe significant correlations between tract integrity (mean FA of thalamocortical tracts connected to the MDn) and the volumes of the connected cortical regions, suggesting that reduced tract integrity was more closely linked to thalamic nuclei atrophy rather than cortical atrophy in these areas.

When assessing the microstructural integrity of thalamic nuclei, MS patients showed significantly higher MD than controls in all four nuclei. Specifically, the posterior nuclei had MD values of 0.0004 ± 0.00005 mm^2^/s in controls and 0.0005 ± 0.00008 mm^2^/s in MS donors (*P* = 0.010); the lateral nuclei showed values of 0.0004 ± 0.00005 mm^2^/s in controls and 0.0005 ± 0.00005 mm^2^/s in MS (*P* = 0.008); the medial nuclei had MD values of 0.0004 ± 0.00005 mm^2^/s in controls and 0.0006 ± 0.00002 mm^2^/s in MS (*P* = 0.001); and the anterior nuclei showed values of 0.0004 ± 0.00005 mm^2^/s in controls and 0.0004 ± 0.00007 mm^2^/s in MS (*P* = 0.023). In contrast, no changes were observed for FA between MS and controls.

### Limited immune cell infiltration in the thalamic MDn in progressive MS

**Fig. 3:**
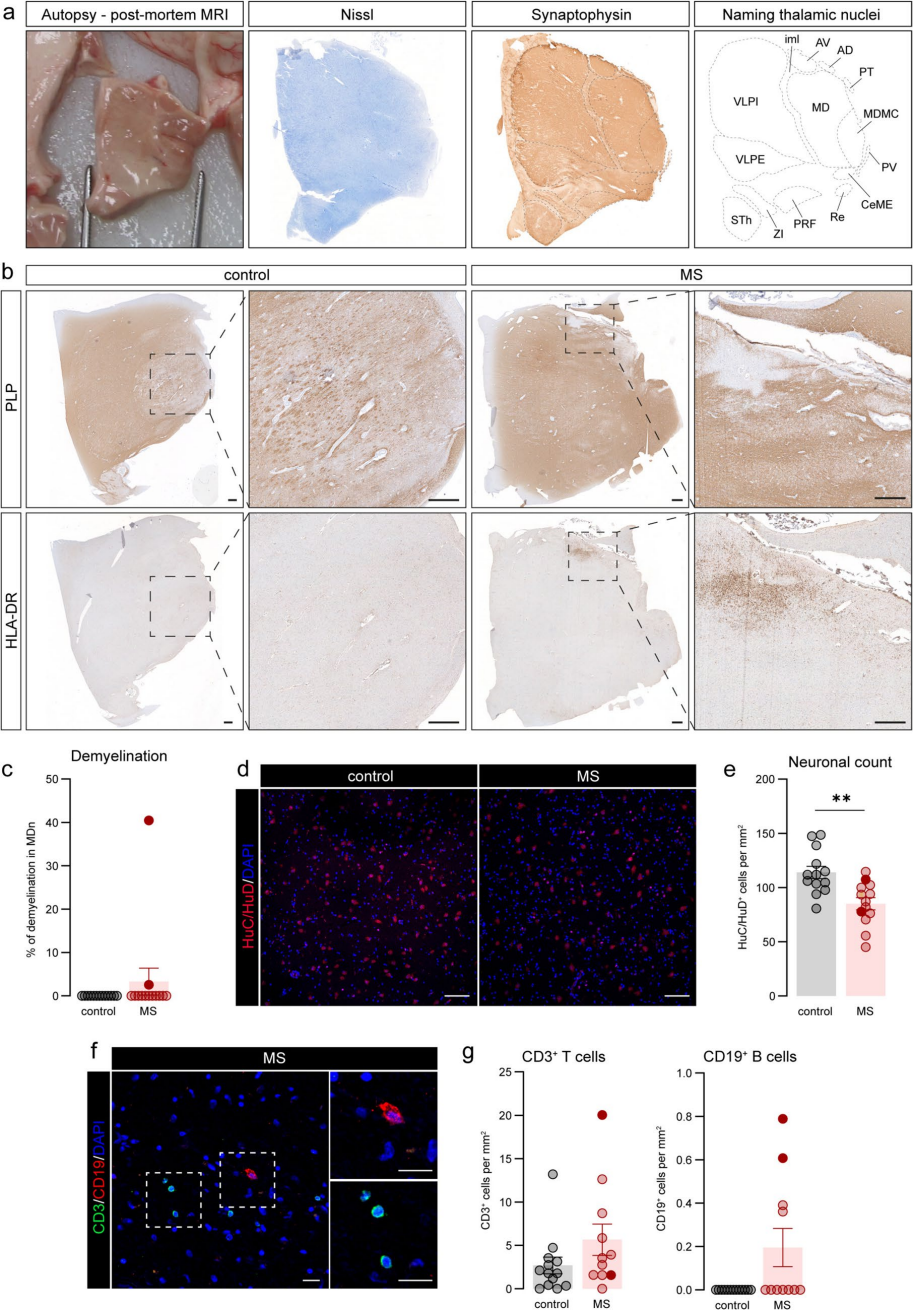
Limited peripheral infiltration and lesions in the MDn of progressive MS donors. **a** Schematic overview for MDn identification based on autopsy and post-mortem MRI, Nissl and Synaptophysin stainings. **b** Representative images displaying PLP and HLA-DR expression of the thalamus of control and MS donors. Zoom-in shows the MDn. **c** Percentage of total demyelination in MDn. **d** Representative images showing HuC/HuD^+^ neurons in the MDn of control and MS subjects. **e** Quantification of HuC/HuD^+^ neurons in the MDn of control and MS donors. **f** Representative staining of CD3^+^ T cells and CD19^+^ B cells in a MS donor. **g** Quantification of CD3^+^ T cells and CD19^+^ B cells in the MDn of control and MS donors. Individual datapoints indicate averaged data from an individual donor, columns and error bars show mean ± SEM; ** *P* < 0.01. Filled datapoints show MS donors with lesions in the MDn. Scale bars = 200 µm (**b**), 100 µm (**d**), 50 µm (**f**). *AD* anterodorsal nucleus, *AV* anteroventral nucleus, *CeME* central medial nucleus, *iml* internal medullary lamina, *MDn* mediodorsal nuclei, *MDMC* mediodorsal nucleus, magnocellular part, *PRF* prerubral field (ruber nucleus), *PT* paratenial nucleus, *PV* paraventricular nucleus, *Re* reuniens nucleus, *STh* subthalamic nucleus, *VLPE* ventral posterolateral nucleus, external part, *VLPI* ventral posterolateral nucleus, internal part, *ZI* zona incerta, *MS* multiple sclerosis

After tissue collection, we first annotated the different thalamic nuclei using the strategy seen in Fig. 3a. Next, to investigate the involvement of local demyelination and peripheral inflammation in thalamic atrophy, we first studied the lesion load of the whole thalamus blocks using bright-field microscopy on PLP^+^ and HLA-DR^+^ stained sections of controls and MS brains (Fig. 3b). We only identified three MS donors that contained demyelinating lesions in the whole thalamus, which were all surrounded by a rim of HLA-DR^+^ cells, indicating the presence of chronic active lesions. From these three MS donors, two donors contained lesions in the MDn (Fig. 3c, Supplementary Fig. 1a). We decided to focus our analysis on this nucleus, as being part of the medial nuclei group and therefore, more atrophic (Fig. 1d). We quantified the number of HuC/HuD^+^ cells in the MDn and observed a modest but significant loss of neurons in MS donors compared to controls (*P* = 0.002) (Fig. 3d, e, Supplementary Fig. 1b). We further studied the infiltration of different immune cells into the parenchyma of the MDn and found that CD3^+^ T cells were equally present in MS and control donors. As expected, CD19^+^ B cells were absent in the MDn of non-neurological controls (Fig. 3f, g). Although the numbers of CD19^+^ B cells were small, we found a significant increase in CD19^+^ cell density in MS compared to control donors (*P* = 0.047) (Supplementary Fig. 1c). Interestingly, the three MS donors with higher numbers of CD19^+^ B cells in the MDn were the ones that contained lesions inside and outside the MDn. Taken together, in our cohort we observed limited peripheral immune cell infiltration in the thalamic MDn.

### Thalamic MDn microglia are morphologically more complex and engulf pre-synapses in progressive MS

**Fig. 4:**
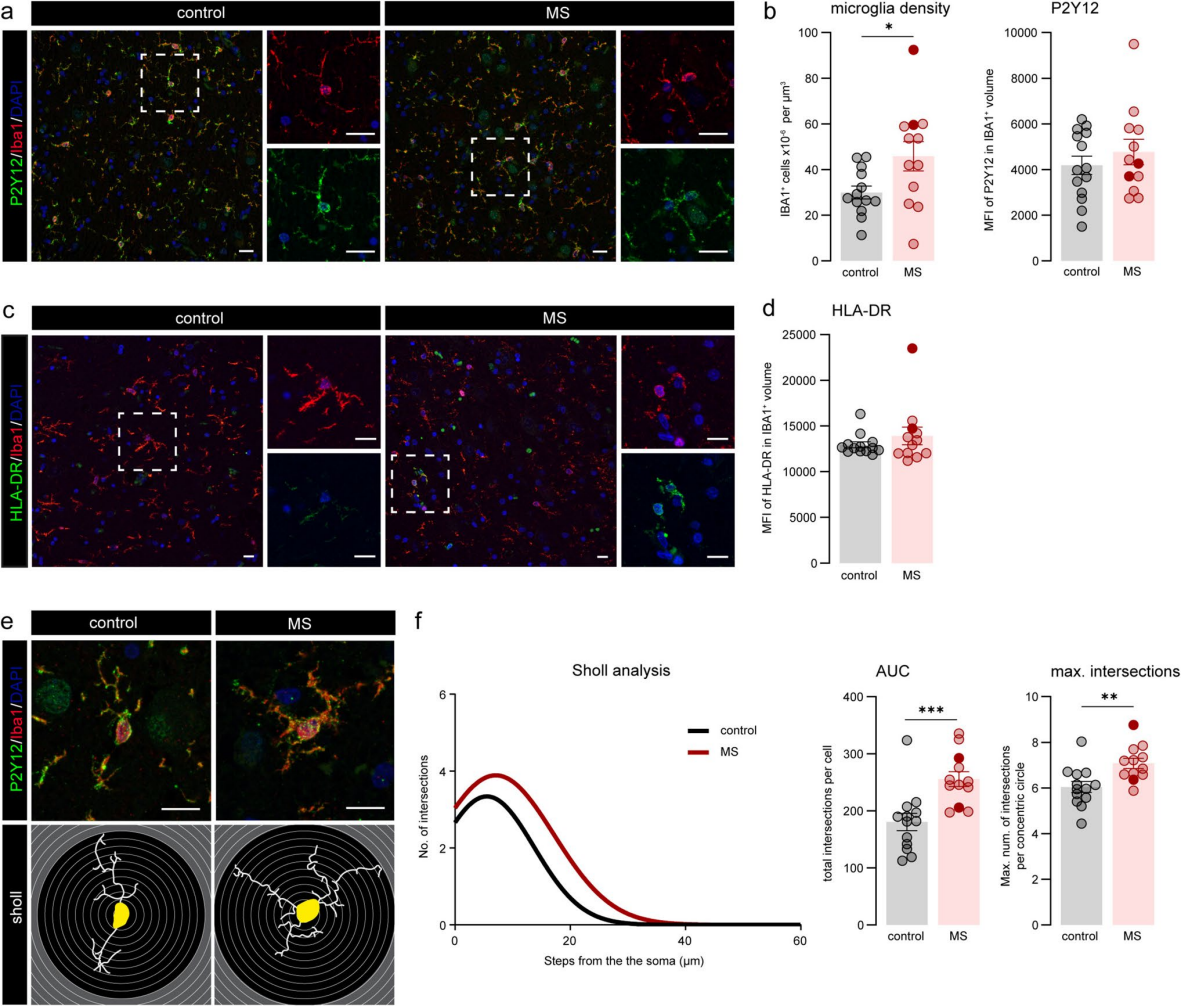
Microglial morphology complexity is increased in progressive MS. **a** Representative stainings showing P2Y12^+^ and IBA1^+^ microglia in MDn of control and progressive MS donors. **b** Quantification of the microglial density and mean fluorescence intensity of P2Y12 in IBA1^+^ microglia. **c** Representative images showing HLA-DR + and IBA1 + microglia in MDn of control and progressive MS donors. **d** Quantification of the mean fluorescence intensity of HLA-DR in IBA1^+^ cells. **e** Images of individual P2Y12^+^ IBA1^+^ microglia in MDn nuclei (top panels) and the corresponding tracing outlines (bottom panels). **f** Sholl-derived measurements. Non-linear curve fit of the average number of microglial branch intersections per 0.3 µm step from the cell soma. Area under the curve (AUC) and maximal number of intersections of microglial cell morphology averaged per donor. Individual datapoints indicate averaged data from an individual donor, columns and error bars show mean ± SEM; * *P* < 0.05, ** *P* < 0.01, *** *P* < 0.001. Filled datapoints show MS donors with lesions in the MDn. Scale bars = 20 µm (**a, c, e**). *MFI* mean fluorescence intensity, *MDn* mediodorsal nucleus, *MS* multiple sclerosis

We next characterized microglia, the resident immune cell of the brain, which were identified as IBA1^+^ P2Y12^+^ cells. Microglial density was significantly higher in progressive MS compared to controls (*P* = 0.038), although there was a high variability between donors (Fig. 4a, b, and Supplementary Fig. 2a). We further quantified the expression of the homeostatic microglia marker P2Y12 on IBA1^+^ microglia. Thalamic microglia in progressive MS and control donors had similar expression levels of this marker (Fig. 4b), indicating a non-reactive microglia profile in the MDn of progressive MS. To further validate this observation, we quantified HLA-DR expression in IBA1^+^ microglia as a marker of microglial reactivity and found comparable levels between control and progressive MS donors (Fig. 4c, d). Interestingly, IBA1^+^ P2Y12^+^ microglia in the MDn were more ramified in MS compared to controls (*P* = 0.0003), evidenced by the increased area under the curve (AUC) of the Sholl analysis [17] (Fig. 4e, f). Microglial morphological analysis also revealed an increase in the maximum number of intersections in progressive MS (*P* = 0.004) (Fig. 4f). Notably, when analyzing microglia from randomly sampled regions across the entire thalamus—without distinguishing between specific thalamic nuclei—we observed a similar phenotype to that of the MDn, although the changes were generally less pronounced (Supplementary Fig. 2b-c).

**Fig. 5:**
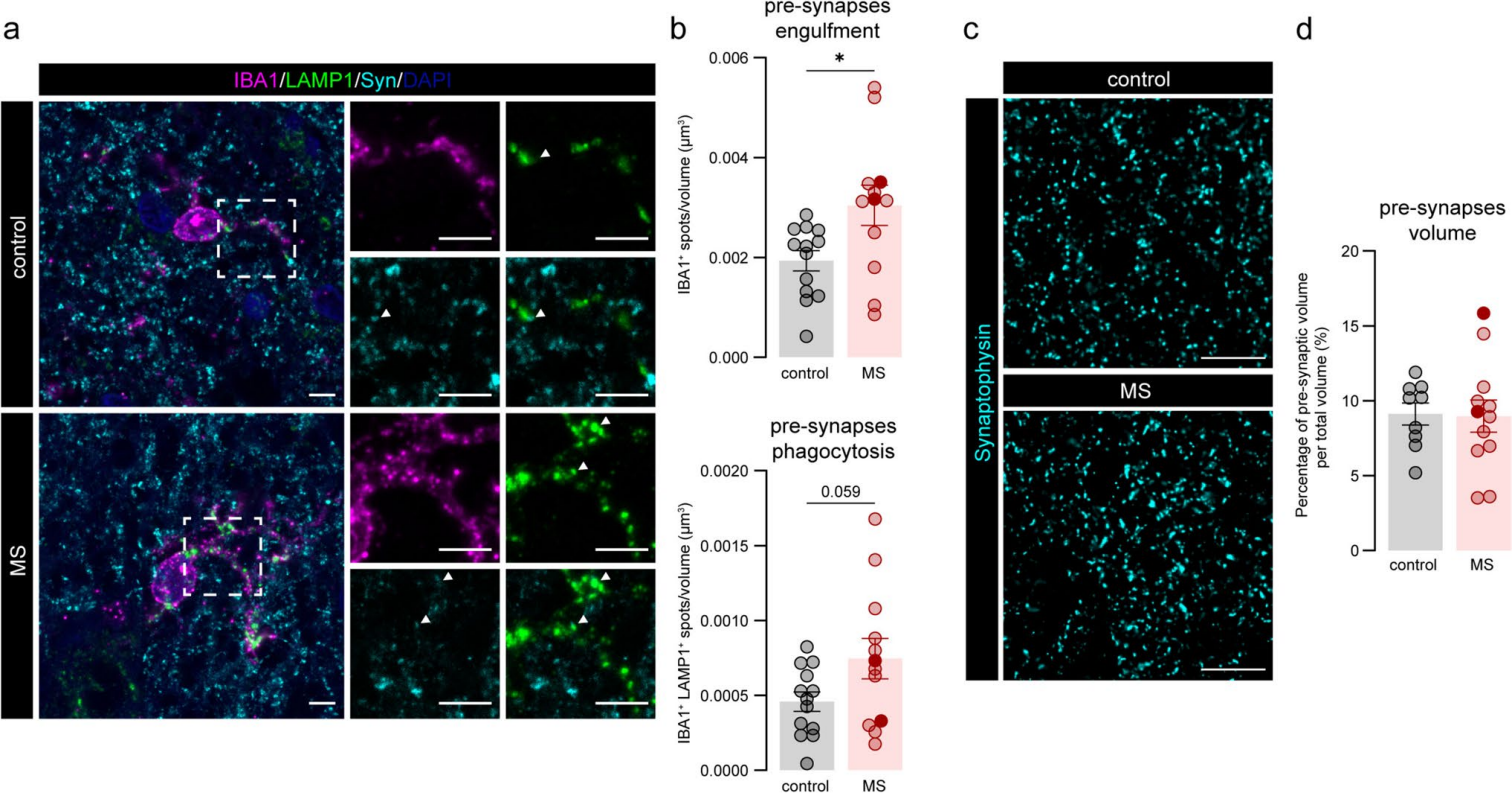
Microglial pre-synapses engulfment is enhanced in progressive MS. **a** Representative images of control and MS sections stained for IBA1 (microglia), LAMP1 (lysosomes) and Synaptophysin (pre-synapses) in the MDn. **b** Quantification of Synaptophysin^+^ spots in IBA1^+^ microglia (pre-synapses engulfment) and in IBA1^+^ LAMP1^+^ microglial lysosomes (pre-synapses phagocytosis). **c** Representatives images of synaptophysin in the MDn. **d** Percentage of pre-synaptic volume in control and MS donors. Individual datapoints indicate averaged data from an individual donor, columns and error bars show mean ± SEM; * *P* < 0.05. Filled datapoints show MS donors with lesions in the MDn. Scale bars = 5 µm (**a**), 10 µm (**c**). *MDn* mediodorsal nucleus; MS: multiple sclerosis

In the lateral geniculate nuclei of MS donors, microglia were previously found to phagocytose presynaptic terminals [52]. We next questioned whether the same was true in the MDn in our cohort. To do so, we quantified the amount of Synaptophysin^+^ structures in LAMP1^+^ lysosomes in IBA1^+^ microglia. Similar to the lateral geniculate nuclei of the thalamus and the MS cortex [48, 52], we found a significant increase of pre-synapses within IBA1^+^ microglia (*P* = 0.025) and a trend towards more pre-synapses in LAMP1^+^ lysosomes within IBA1^+^ microglia (*P* = 0.059). Furthermore, we observed an increased lysosomal volume in MS microglia (*P* = 0.045) (Fig. 5a, b, Supplementary Fig. 3a). However, when we quantified pre-synaptic density in the MDn by counting the presence of Synaptophysin^+^ spots, we did not observe a loss in total pre-synaptic density nor the volume occupied by pre-synapses in MS donors (Fig. 5c, d, Supplementary Fig. 3b). These results show that in the MS thalamic MDn, regardless of the lack of demyelination, microglia have an impaired phenotype with increased density, morphological complexity and propensity to phagocytose synapses.

### Thalamic atrophy and disconnection correlate with microglial phenotype

**Fig. 6:**
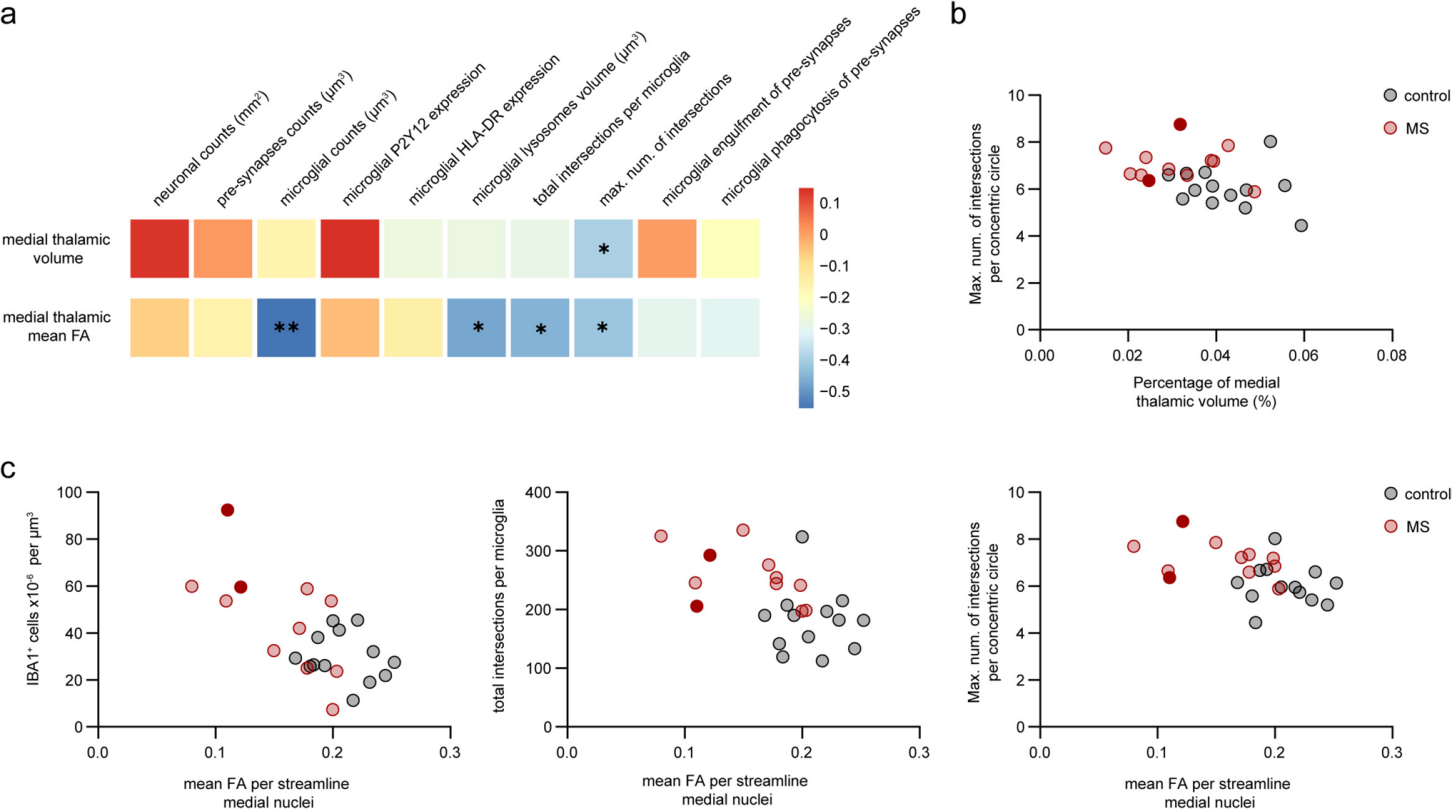
Thalamic atrophy and disconnection correlates with microglial changes. **a** Heatmap indicating partial correlation values between MRI variables (medial thalamic volume and medial thalamic mean FA) and immunohistochemistry variables. Colour key represents positive (red) and negative (blue) correlations. * *P* < 0.05, ** *P* < 0.01. **b** Scatter plot of correlation between microglial morphology and medial thalamic volume. **c** Scatter plots of specific correlation between microglial characteristics and medial mean FA. Individual datapoints indicate averaged data from an individual donor. Filled datapoints show MS donors with lesions in the MDn. *FA* fractional anisotropy, *GM* grey matter, *MDn* mediodorsal nucleus, *MS* multiple sclerosis

Next, we correlated certain histopathological features with MRI measurements from the medial thalamic nuclei (Fig. 6a). Medial thalamic volume was negatively correlated to the average maximum number of microglial ramifications (*r* = − 0.396, *P* = 0.049) (Fig. 6b), indicating that local microstructural changes in the thalamus like a more complex microglia morphology may potentially contribute to the observed thalamic atrophy in MS. Interestingly, mean FA-weighted connectivity of the medial thalamic nuclei did not correlate with neuronal counts or pre-synaptic counts, but did correlate negatively with microglial counts (*r* = − 0.553, *P* = 0.005), the volume of microglial lysosomes, (*r* = − 0.473, *P* = 0.020) and microglia morphology (total number of intersections: *r* = − 0.451, *P* = 0.027; maximum number of intersections: *r* = − 0.417, *P* = 0.043) (Fig. 6a, c). Hence, the decreased integrity of structural networks in MS is associated with this observed microglial phenotype within the thalamus.

## Discussion

By combining post-mortem MRI with immunohistochemistry on a cohort of MS donors and controls, we uncovered that thalamic atrophy in progressive MS is associated with structural disconnection in the form of tract-specific WM damage throughout the brain and microglial changes within the thalamus, but not with thalamic demyelination. Our analysis revealed distinct vulnerability patterns in MS, with the medial and posterior thalamic nuclei exhibiting greater alterations, which indicates a possible contribution of CSF-mediated factors to thalamic atrophy. Furthermore, we found thalamic disconnection to be associated with microglial changes within the MDn, including changes in microglial counts and microglial morphology.

Our cohort did not show frequent thalamic lesions by IHC, despite being near periventricular WM areas that are known as hotspots for MS lesions, likely due to the presence of immune cells and inflammatory mediators in the CSF [1, 7, 18, 21, 26, 50]. Only three MS donors of our cohort presented periventricular thalamic lesions. In addition, we observed a modest neuronal loss in MS, but no overall decrease of pre-synaptic density in the MDn, This limited presence of classical MS pathology may reflect the nature of our donor cohort, which primarily included individuals with long-standing, progressive MS and relatively low thalamic inflammatory activity. In contrast, the three donors with thalamic lesions were younger and had shorter disease duration, suggesting a more active and aggressive disease course. This aligns with previous studies that focused on similar donor profiles and reported more robust pathological changes [50]. These findings reflect the known pathological heterogeneity within progressive MS. While other studies have reported more extensive thalamic demyelination, inflammation, and neuronal loss, our cohort was characterized by long disease duration and low inflammatory activity [8, 28]. Rather than being a limitation, this highlights that substantial thalamic atrophy and microglial alterations can occur even in the absence of active inflammation, pointing to alternative mechanisms such as Wallerian degeneration or CSF-mediated processes. Despite the lack of thalamic demyelination or synaptic loss in our cohort, we did find more pronounced atrophy in the medial and posterior nuclei, which are in closest proximity to the CSF. This suggests that while CSF proximity may not be sufficient to drive lesion formation in the thalamus, it could still contribute to neuronal loss, or tissue damage and atrophy through diffuse inflammatory signals or modulation of microglial behavior. In line with this, our analysis of microglia across the entire thalamus revealed a similar, though less pronounced, phenotype compared to the MDn, further supporting the idea that CSF proximity may amplify microglial responses and tissue vulnerability specifically in medial regions of the thalamus.

Thalamic volume and thalamic nuclear volumes by MRI were related to structural disconnection in the form of reduced mean FA within tracts. Our results are consistent with previous studies that show that thalamic atrophy in MS is influenced by damage to WM tracts, proposedly through mechanisms of Wallerian degeneration, highlighting a potential relationship between microstructural changes in connectivity and neurodegeneration in the thalamus [29, 41, 51]. This disconnection-driven neurodegeneration may be more severe in areas already under stress due to contact with the CSF, i.e. medial and posterior nuclei. Whether specific CSF factors contribute to these effects remains to be determined. Nonetheless, the morphological changes observed in thalamic MDn microglia in this study bear resemblance to our previous observations in cortical microglia [48]. In that earlier study, altered cortical microglia phenotypes were proposed to be influenced by inflammatory factors present in the CSF of the subarachnoid space. In this present study, thalamic microglia appeared morphologically more complex and showed evidence of pre-synaptic engulfment in MS, features reminiscent of those seen in the cortex of progressive MS donors [48]. Although we saw limited peripheral immune infiltration in the MDn, we cannot rule out the possibility that CSF-related inflammatory factors may play a role in shaping these thalamic microglial changes. The number of phagocytosed pre-synapses by thalamic microglia was relatively small, which could explain the absence of an overall decrease in pre-synaptic density in the MDn. Recently, presence of B cell aggregates in perivascular spaces close to the ependymal layer of the thalamus have been linked to thalamic demyelination and neurodegeneration in MS [28]. In our cohort, likely due to its low inflammatory status, we did not detect these B cell aggregates, which might explain why we did not observe an overall demyelination nor synapse loss.

Here, we also showed an association between reduced WM tract connectivity to the medial nuclei of the thalamus and microglial alterations in the MDn, such as microglial counts and morphology. This is in line with previous animal research where experimentally-induced neocortical lesions lead to thalamic degeneration and altered microglial responses [44]. Together, we hypothesize that changes reflected by low FA along fibre tracts contribute to neurodegeneration and microglial alterations within the thalamus. Given that thalamic nuclei closer to the third ventricle are more vulnerable to damage [24], we suggest a two-hit phenomenon driving thalamic atrophy in MS based on Wallerian degeneration and impaired WM connectivity and, secondly, with local CSF-factors and modified microglial responses. While thalamic nuclei, particularly those in medial and posterior groups, exhibited clear signs of atrophy and altered microglial phenotype, cortical regions structurally connected to the MDn did not show evidence of atrophy in our post-mortem cohort. This absence of cortical volume loss may partly reflect increased segmentation noise in post-mortem tissue affecting both MS and control donors, reducing sensitivity to detect subtle cortical changes. Alternatively, it suggests that the thalamus may be more vulnerable to dual mechanisms proposed in the two-hit model—namely, Wallerian degeneration and CSF-mediated toxicity—whereas connected cortical regions may be less exposed. This interpretation aligns with our previous in vivo observations [24]. Furthermore, while reduced FA in thalamocortical tracts was associated with reduced MD nuclei volume, we did not observe a clear relationship between tract integrity and cortical atrophy in the connected regions, suggesting that degeneration may be more localized to vulnerable network hubs such as the thalamus, rather than uniformly extending to connected cortical areas. Future studies combining histopathology with longitudinal in vivo imaging could better capture network-driven atrophy dynamics in MS.

One potential limitation of our study is that while the sample size was sufficient for histopathological analysis, it was relatively small for neuroimaging analysis, which may have reduced the statistical power of our study. In addition, due to the handling of post-mortem tissue during autopsy, the collected thalamic blocks did not contain all thalamic nuclei and we were restricted to the MDn only. This hindered our ability to fully explore the pathological mechanisms underlying the differential vulnerability of thalamic nuclei and make direct comparisons between the thalamic groups. Furthermore, our MS cohort consisted predominantly of female donors, with only one male participant. Although MS is more common in females, male patients are often more susceptible to neurodegeneration and progression [2, 36]. As such, this imbalance may limit the generalizability of our findings across sexes. Lastly, while we assessed the integrity of thalamocortical white matter tracts using diffusion MRI, histological examination of these tracts was not possible due to the limited availability of connected white matter tissue. Furthermore, diffusion MRI does not allow differentiation between afferent and efferent fibers, which could have important functional implications. Future studies incorporating histological and pathway-specific analyses could provide additional insights into the cellular mechanisms underlying tract-related thalamic degeneration, and the specific contributions of afferent input loss. On the other hand, our study has the strength of combining post-mortem MRI and immunohistochemistry which enabled us to investigate and correlate multiple aspects of pathology like thalamic atrophy and local microstructural abnormalities. Finally, the clinical history of these subjects remains unclear, and future work with more extensive clinical scores and treatment history would enrich such a dataset further.

In summary, this study reveals that thalamic atrophy in MS is primarily related to the disconnection of thalamo-cortical tracts and associates with local microglial changes, but not thalamic demyelination. In addition, medial and posterior thalamic nuclei were more severely affected in MS, implicating a role for contact with the CSF and support the concept of a two-hit phenomenon in thalamic atrophy in MS. Future studies are now required to investigate whether slowing down white matter lesions and microglial changes would be feasible to reduce thalamic neurodegeneration in MS. Moreover, characterizing cohorts across different inflammatory profiles will be essential to fully understand the mechanisms and variability of thalamic damage in MS.

## Supplementary Information

Below is the link to the electronic supplementary material.

Supplementary file1 (PDF 414 KB)

## Data Availability

The data that support the findings of this study are available from the corresponding author upon reasonable request.
