# Supplementary material for: Thalamic atrophy in multiple sclerosis is associated with tract disconnection and altered microglia
Source: Acta Neuropathol. 2025 May 28;149(1):52. doi: 10.1007/s00401-025-02893-4 (PMC12119693; doi:10.1007/s00401-025-02893-4)
Supplement: Supplementary file 1 — Supplementary file1 (PDF 414 KB) [file 401_2025_2893_MOESM1_ESM.pdf]

Supplementary information

Thalamic atrophy in multiple sclerosis is associated with tract disconnection and altered microglia

Carla Rodriguez-Mogeda[1,2,†], Ismail Koubiyr[2,3,†], Stefanos E. Prouskas[2,3], Margarita Georgallidou[3], Susanne M. A. van der Pol[1,2], Rosalia Franco Fernandez[1], Yvon Galis – de Graaf[3], Ysbrand D. van der Werf[3], Laura E. Jonkman[3], Geert J. Schenk[2,3], Frederik Barkhof[2,4,5], Hanneke E. Hulst[3,6], Maarten E. Witte[1,2], Menno M. Schoonheim[2,3,#] and Helga E. de Vries[1,2,#]

[1] Amsterdam UMC, Vrije Universiteit Amsterdam, Molecular Cell Biology and Immunology, De Boelelaan 1117, Amsterdam, The Netherlands

[2] MS Center Amsterdam, Amsterdam UMC location VUmc, Amsterdam Neurosciences, Amsterdam, The Netherlands.

[3] Amsterdam UMC, Vrije Universiteit Amsterdam, Anatomy and Neurosciences, De Boelelaan 1117, Amsterdam, The Netherlands

[4] Amsterdam UMC, Vrije Universiteit Amsterdam, Radiology and Nuclear Medicine, De Boelelaan 1117, Amsterdam, The Netherlands

[5] Queen Square Institute of Neurology and Centre for Medical Image Computing, University College London, London WC1E 6BT, United Kingdom

[6] Institute of Psychology, Health, Medical and Neuropsychology Unit, Leiden University, Wassenaarseweg 52, Leiden, The Netherlands

[†]These authors contributed equally to this work.

[#] These authors are shared senior authors.

Correspondence to: Menno M. Schoonheim (E-mail: m.schoonheim@amsterdamumc.nl)

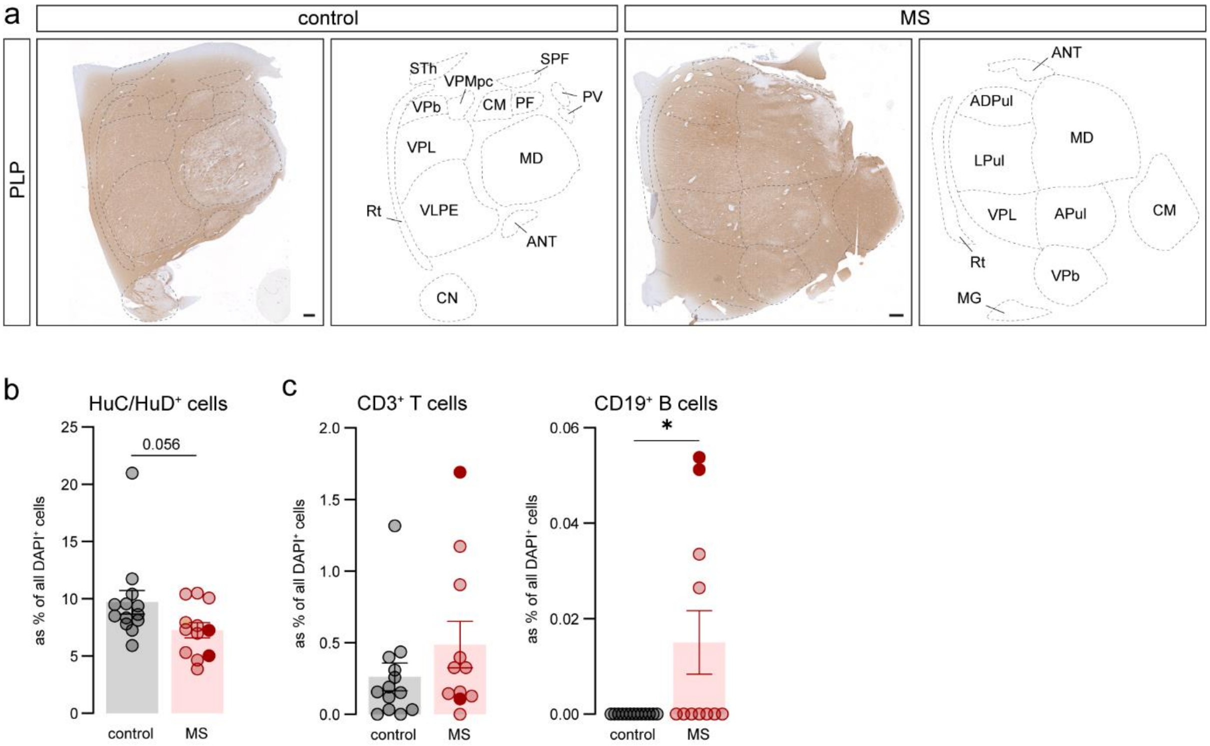

Supplementary Fig. 1

a. Schematic overview for MDn identification from insets in Fig. 3b. Scale bars = 200 µm. **b**. Percentage of HuC/HuD^{+} neurons in the MDn of control and MS donors. **c.** Percentage of CD3^{+} T cell and CD19^{+} B cell in the MDn of control and MS donors. Individual datapoints indicate averaged data from an individual donor, columns and error bars show mean ± SEM. * $P < 0.05$. Filled datapoints show MS donors with lesions in the MDn. ADPul: anterodorsal pulvinar nucleus; ANT: anterior nucleus; APul: anterior pulvinar nucleus; CM: central medial nucleus of the rostral intralaminar complex; CN: caudate nucleus; MDn: mediodorsal nucleus; MG: medial geniculate nucleus; LPul: lateral pulvinar nucleus; PF: parafascicular nucleus; PV: paraventricular nucleus; Rt: reticular nucleus; SPF: subparafascicular nucleus; STh: subthalamic nucleus; VLP: ventral posterolateral nucleus; VLPE: ventral posterolateral nucleus, external part. VPMpc: ventral posteromedial nucleus, parvocellular part; VPb: basal ventroposterior nucleus; MS: multiple sclerosis.

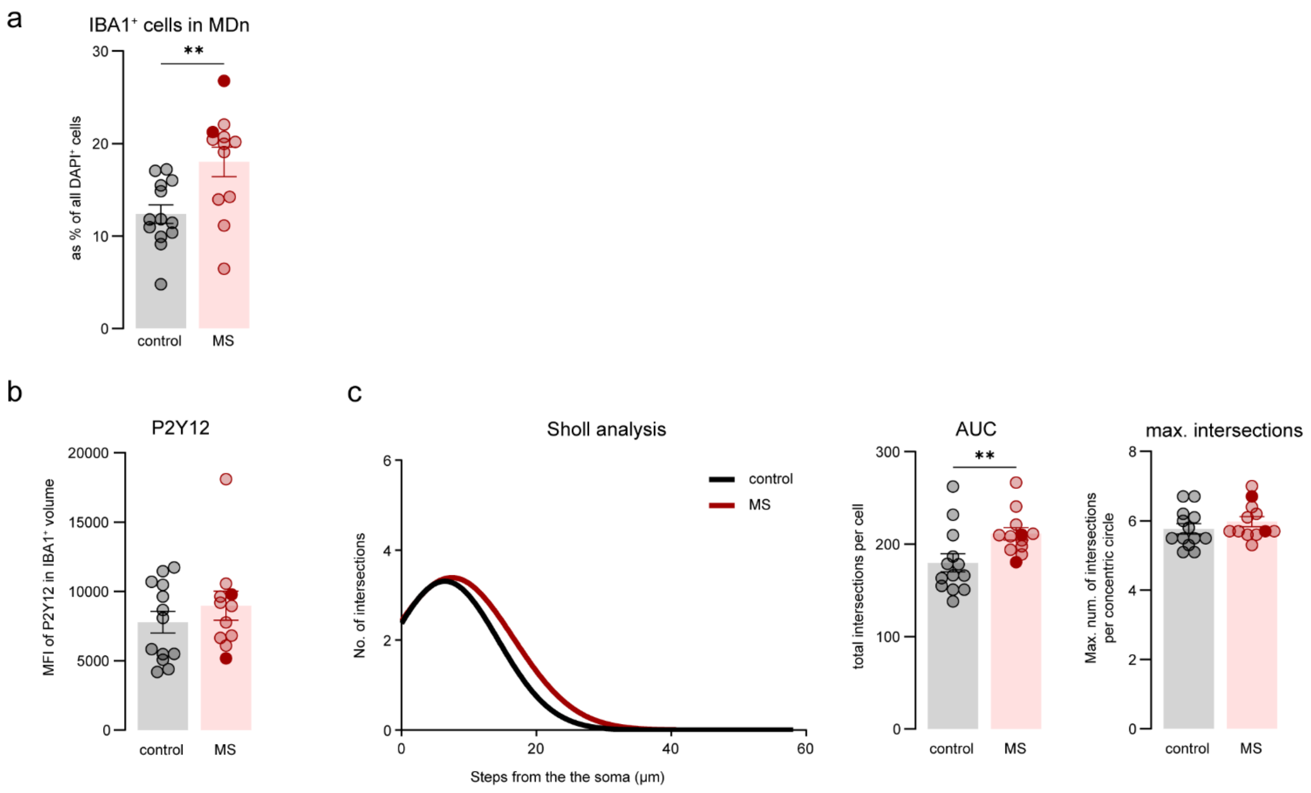

Supplementary Fig. 2

a. Percentage of IBA1^{+} cells in MDn of control and MS donors. **b.** Quantification of mean fluorescence intensity of P2Y12 in IBA1^{+} microglia of the whole thalamus of control and MS donors. **c**. Sholl-derived measurements of microglia of the whole thalamus. Non-linear curve fit of the average number of microglial branch intersections per 0.3 µm step from the cell soma. Area under the curve (AUC) and maximal number of intersections of microglial cell morphology averaged per donor. Individual datapoints indicate averaged data from an individual donor, columns and error bars show mean ± SEM; ** $P < 0.01$. Filled datapoints show MS donors with lesions in the MDn. MFI: mean fluorescence intensity; MDn: mediodorsal nucleus; MS: multiple sclerosis.

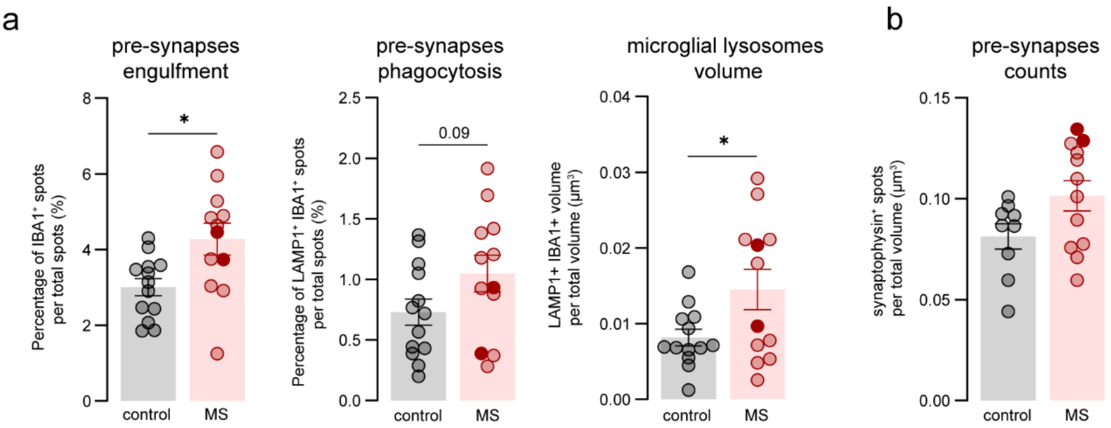

Supplementary Fig. 3

a. Percentage of Synaptophysin$^+$ spots that are located in IBA1$^+$ microglia and LAMP1$^+$ IBA1$^+$ microglial lysosomes in the MDn of control and MS donors and volume of microglial lysosomes per total volume of the MDn. **b.** Total Synaptophysin$^+$ spot density in MDn of control and MS donors. Individual datapoints indicate averaged data from an individual donor, columns and error bars show mean ± SEM; * P < 0.05. Filled datapoints show MS donors with lesions in the MDn. MDn: mediodorsal nucleus; MS: multiple sclerosis.
